# Safety and efficacy of flexible and navigable suction combined with retrograde flexible ureteroscopy in the management of infectious stones

**DOI:** 10.1186/s12879-025-11508-y

**Published:** 2025-08-28

**Authors:** Deheng Cui, Qinghong Ma, Guangzhi Wang, Jianbin Luo, Guoqiang Chen

**Affiliations:** Department of Urology, The Second Hospital of Longyan, Longyan, 364000 Fujian China

**Keywords:** Flexible and navigable suction, Retrograde intrarenal surgery, Infectious stones

## Abstract

**Objective:**

To compare the safety and efficacy of flexible and navigable suction (FANS) ureteral access sheath-assisted retrograde intrarenal surgery (FANS-RIRS) for infectious versus non-infectious stones.

**Methods:**

A retrospective study of 274 patients treated with FANS-RIRS (November 2023–December 2024). Patients were stratified postoperatively into groups based on stone composition. The infectious stone group (*n* = 61) comprised stones containing at least one of the following components: magnesium ammonium phosphate hexahydrate (struvite), carbonate apatite, or ammonium urate, either as pure or mixed compositions. The non-infectious stone group (*n* = 213) included other stone types such as metabolic stones, those associated with genetic abnormalities, and drug-induced stones. Outcomes included infectious complications (SIRS/sepsis), stone-free rate (SFR), operative time, and postoperative complications. Statistical analysis used t-tests, Mann-Whitney U, and chi-square tests.

**Results:**

No sepsis occurred in either group. Immediate SFR was comparable (90.2% vs. 92.1%, *p* = 0.638), with residual stones in infectious cases primarily in lower-pole calyces or diverticula. Infectious stones showed higher preoperative urine culture positivity (32.8% vs. 20.6%, *p* = 0.046), but no differences in operative time, hospital stay, or complications.

**Conclusion:**

FANS-RIRS for infectious stones demonstrated comparable postoperative infection rates to non-infectious stones, proving safe and effective. However, postoperative antimicrobial stewardship and regular surveillance remained paramount for infectious stone management.

## Introduction

Urinary stones ranked third among urological diseases in terms of incidence and socioeconomic burden, with a high prevalence observed globally [1]. Infectious stones, in particular, were more likely to present clinical symptoms such as flank pain (approximately 70%), hematuria (approximately 18%), and infection (approximately 27%) [2]. These characteristics were closely associated with the pathogenesis of infectious stones. Pathogenic bacteria in urine hydrolyze urea to produce ammonia, alkalinizing the urine and promoting rapid crystallization of magnesium ammonium phosphate hexahydrate (struvite), carbonate apatite, and ammonium urate [3]. Approximately 35% of infectious stones yield positive urine or stone cultures, with *Proteus mirabilis* and *Escherichia coli* being the predominant isolates [4]. Consequently, the therapeutic principles for infectious stones focus on complete stone clearance, infection control, and renal function preservation.

Current management strategies include pharmacological therapy and surgical intervention. Pharmacological approaches, primarily used as postoperative adjuvants, are moderately effective but costly. In recent years, retrograde intrarenal surgery (RIRS) has expanded in scope due to advancements in lithotripsy devices, improving efficiency and safety [5]. However, infection remains a critical postoperative complication. Previous studies on RIRS identified infectious stones as a high-risk factor for postoperative infections due to the high bacterial burden within stones and pelvic urine [6]. Notably, a 2024 international multicenter prospective randomized controlled trial conducted by Professor Guohua Zeng demonstrated that RIRS combined with a flexible and navigable suction achieved lower complication and infection rates and higher stone-free rates (SFR) compared to traditional ureteroscope sheaths in renal stone management [7]. However, this study did not analyze infectious stones due to the preoperative challenges in accurately determining stone composition. Whether negative-pressure RIRS offers similar low-infection advantages for infectious versus non-infectious stones, or whether operative time and SFR differ between groups, has not been previously reported. Therefore, we conducted a retrospective analysis of our institutional data, categorizing cases by postoperative stone composition, to evaluate the safety and efficacy of FANS-RIRS for infectious stones, summarize clinical insights, and promote broader adoption of this technique.

## Materials and methods

The study was approved by the Ethics Committee of the Second Hospital of Longyan City, Fujian Province, and written informed consent was obtained from all patients for the use of their data in this research. The study adhered to the ethical standards of the 1964 Declaration of Helsinki and its subsequent amendments. Clinical data were retrospectively collected from patients admitted to the Department of Urology at Longyan Second Hospital between November 2023 and December 2024. Inclusion criteria were: (1) CT-confirmed renal and/or ureteral stones; (2) total stone diameter ≤ 3 cm; (3) treatment with S- RIRS; (4) postoperative stone composition analysis. Exclusion criteria included: (1) simultaneous bilateral procedures; (2) concurrent additional surgeries; (3) uncontrolled urinary tract infections (Table 1).

**Table 1:** Number of excluded samples and reasons for exclusion

Cause of exclusion	Number
Bilateral RIRS	117
RIRS combined with PCNL	13
RIRS combined with TURP	1
RIRS combined with bladder calculi lithotripsy	3
RIRS combined with urethral stricture dilatation	1
Uncontrolled urinary tract infection	2
Total	137

Patients were stratified postoperatively into groups based on stone composition. The infectious stone group (*n* = 61) comprised stones containing at least one of the following components: magnesium ammonium phosphate hexahydrate (struvite), carbonate apatite, or ammonium urate, either as pure or mixed compositions [8]. The non-infectious stone group (*n* = 213) included other stone types such as metabolic stones, those associated with genetic abnormalities, and drug-induced stones. A non-contrast computed tomography (NCCT) scan were performed in all patients before the operation. Stone size and density were measured using identical software. For patients with positive preoperative urine culture, targeted antibiotics were administered for 4 days, followed by a repeat culture. If sterile, surgery proceeded, with postoperative antibiotics for 7 days. If still positive, antibiotics were extended to 7 days total, with a second culture; only sterile cases proceeded to surgery (postoperative antibiotics for 10 days). Persistent bacteriuria after 7 days led to exclusion. Patients with negative preoperative culture received a single preoperative dose of levofloxacin (500 mg) and 24-hour postoperative prophylaxis. All procedures were performed by one designated experienced surgeon (≥ 100 procedures per year in FANS-RIRS).

Baseline patient data included age, sex, comorbidities (hypertension/diabetes mellitus), and initial urine culture results. Stone-related parameters encompassed hydronephrosis, stone diameter, CT attenuation values, laterality, location, stone number, and preoperative double-J stent placement. Intra- and postoperative data were recorded, including UAS size, ureteroscope caliber, ureteral stricture, ureteral injury, operative time, SFR, postoperative complications, and stone composition. Complications were graded according to the Clavien-Dindo classification. All data were independently collected by two urologists and cross-checked for consistency.

### Endpoints

The primary endpoint was infectious complications, defined as systemic inflammatory response syndrome (SIRS), sepsis, and septic shock. Secondary endpoints included SFR, operative time, and postoperative complications.

### Definitions

SIRS was diagnosed if patients met at least two of the following criteria: abnormal body temperature (> 38 °C or < 36 °C), tachycardia (heart rate > 90 beats/min), tachypnea (respiratory rate > 20 breaths/min or PaCO₂ <32 mmHg), or leukocyte count abnormalities (> 12,000/µL or < 4,000/µL). Sepsis was defined as infection-induced life-threatening organ dysfunction, characterized by either a ≥ 2-point increase in the Sequential Organ Failure Assessment (SOFA) score from baseline or fulfillment of ≥ 2 quick SOFA (qSOFA) criteria during rapid screening (respiratory rate ≥ 22 breaths/min, altered mentation, or systolic blood pressure ≤ 100 mmHg) [9]. Septic shock was diagnosed when sepsis coexisted with persistent hypotension (mean arterial pressure < 65 mmHg requiring vasopressor support), serum lactate > 2 mmol/L, and inadequate response to fluid resuscitation [9].

SFR was defined as the absence of visible stones or residual fragments ≤ 2 mm. Kidney-ureter-bladder X-ray (KUB) was performed within 24 h postoperatively to evaluate SFR. Low-dose NCCT with a 2-mm section thickness was obtained for all patients at the 3-month follow-up to evaluate the final stone-free status. On NCCT, stone free was defined as the absence of all detectable stones, including dust-like particles, or residual fragments ≤ 2 mm in any renal calyx, pelvis, or ureter.

### Surgical procedure

**Fig. 1:**
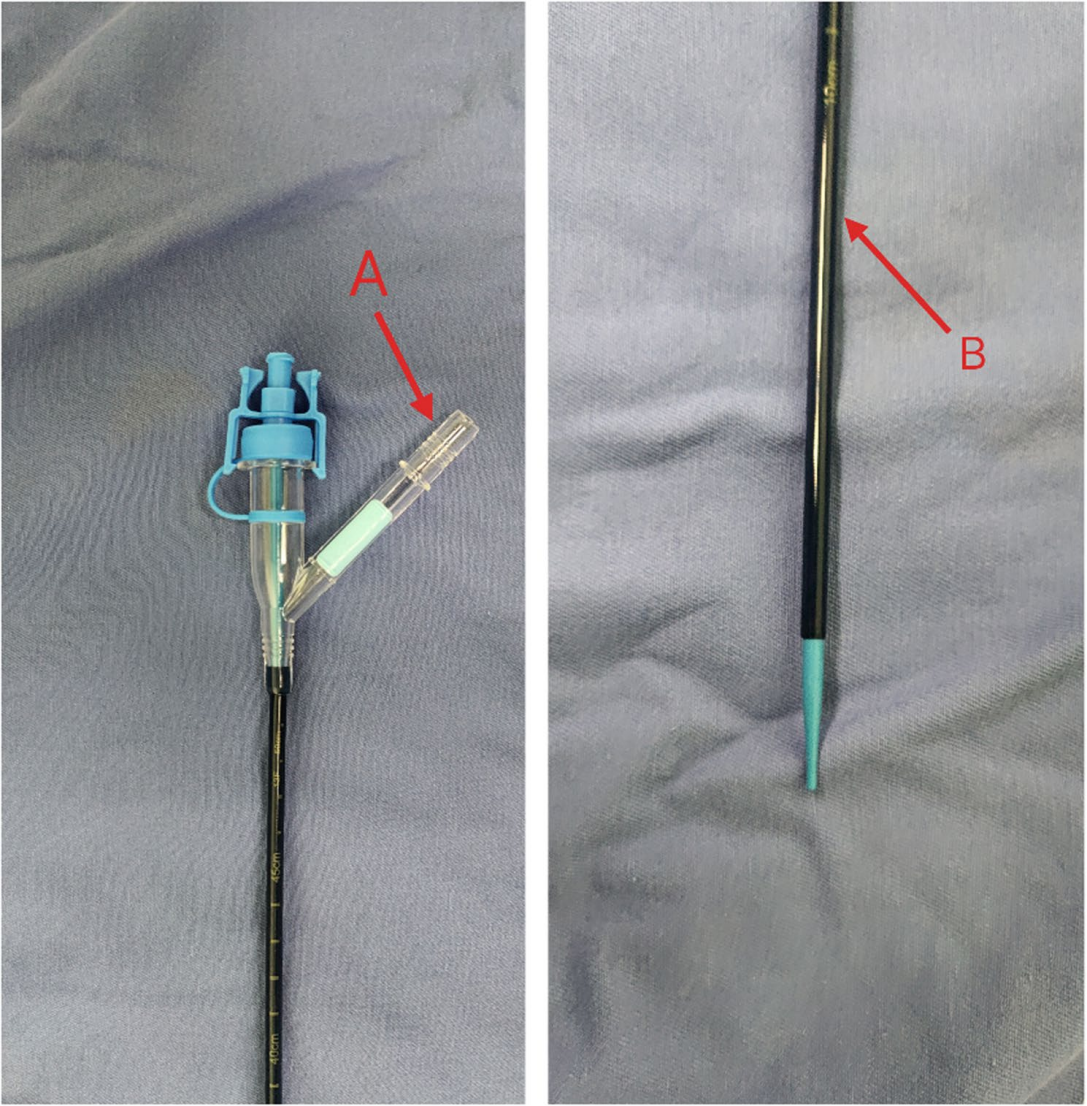
Tip bendable suction ureteral access sheath; (red A is the negative pressure suction connection port, red B is bendable tip)

All procedures were performed under general anesthesia with a lithotomy position. Initial urethral and ureteral access was established using a rigid ureteroscope (8/9.8 F). An 8.5 F–7.5 F disposable flexible ureteroscope was used based on the UAS size: an 8.5 F scope for a 12/14 F UAS and a 7.5 F scope for an 11/13 F UAS (Fig. 1). The end of the UAS was connected to a closed suction system maintaining negative pressure at 0.02–0.04 MPa. A single-use digital flexible ureteroscope (YVD™,China) was advanced through the UAS, with continuous irrigation maintained at 50–120 mL/min using a pressure-controlled pump system. Lithotripsy was performed using a 200 μm holmium: YAG laser fiber (Lumenis^®^) with energy settings (0.6–0.8 J/pulse, 20–30 Hz frequency). Systematic stone fragmentation and dusting were conducted under endoscopic visualization. The deflectable tip of the UAS was positioned to facilitate access to targeted calyces, enabling efficient retrieval of residual fragments through combined suction techniques. Ureteral injuries were visually assessed and classified according to the endoscopic classification of lesions when the UAS was removed along with the ureteroscope. A 6 F indwelling double-J stent was placed for 1–2 weeks if no ureteral injury occurred.

Blood routine and serum procalcitonin levels were measured within 2 h after the operation. Stone composition was analyzed using an automated infrared spectral analysis system (Model LIIR-20, Lambda Scientific Instruments Co., Ltd.).

#### Statistical analysis

The normality of continuous variables was first assessed using Shapiro-Wilk tests, while homogeneity of variances was verified through Levene’s test. When these assumptions were met, an independent samples t-test was employed, with results reported as mean ± standard deviation. For datasets violating parametric assumptions (non-normal distribution or unequal variances), non-parametric analyses were conducted using the Mann-Whitney U test, with findings expressed as median values with interquartile ranges (IQR). Categorical variables were summarized as frequency counts and percentages (n, %). The Chi-square test was applied for nominal categorical comparisons, whereas the Mann-Whitney U test was reserved for ordinal categorical variables. All statistical tests were two-tailed, with α = 0.05 defining statistical significance. Analyses were performed using SPSS software (version 27.0; IBM Corp), maintaining a significance threshold of *p* < 0.05 throughout the study.

## Results

### Preoperative data

No significant differences were observed between the two groups in baseline characteristics, including age, gender, or comorbidities (hypertension/diabetes mellitus) (Table 2). Among the entire cohort, 64 patients (23.4%, 64/274) had positive preoperative urine cultures. A statistically higher proportion of positive cultures was identified in the infection group compared to the non-infection group (32.8% vs. 20.6%, *p* = 0.046). Detailed bacterial distributions are presented in Table 3. In the infection group, *Escherichia coli* constituted the predominant pathogen (35% of isolates), with urease-producing bacteria accounting for 15%. Similarly, *E. coli* was most frequently isolated in the non-infection group (27.3%), though urease-producing strains were rare (0.9%). Analysis of stone parameters revealed comparable profiles between groups for diameter, CT density (Hounsfield units), laterality (unilateral/bilateral), anatomical location, and stone type. However, the severity grading of hydronephrosis differed significantly (*p* = 0.037). Specifically, among patients with renal stones, the distribution in the infectious vs. non-infectious groups was as follows: renal pelvis (9.1% vs. 22.0%), upper calyces (18.2% vs. 20.0%), middle calyces (21.2% vs. 25.0%), lower calyces (21.2% vs. 13.0%), and multiple locations (30.3% vs. 20.0%). Statistical analysis showed no significant difference in stone location between the two groups (*p* = 0.323).

**Table 2:** Demographical characteristics and clinical data of the patients S-UAS, tip bendable Suction ureteral access sheath;

Variables	Infection group(*n* = 61)	Non-infection group(*n* = 213)	*P*-value
Age(year)	53.69 ± 12.40	53.09 ± 11.02	0.954
Gender, n(%)			0.121
Male	30(49.2)	129(60.3)	
Female	31(50.8)	85(39.7)	
Laterality, n(%)			0.308
left	30(49.2)	121(56.5)	
Right	31(50.8)	93(43.5)	
Stone type, n(%)			0.294
Single	35(57.4)	106(49.8)	
Multiple	26(42.6)	107()	
Stone location, n(%)			0.672
Renal	33(54.1)	100(47.7)	
Proximal ureter	6(9.8)	25(11.7)	
Multiple	22(36.1)	87(40.7)	
Stone diameter(mm)	17.52 ± 6.87	15.81 ± 5.99	0.081
CT value of stone(HU)	849.52 ± 310.38	807.53 ± 291.29	0.329
Comorbidities, n(%)			0.787
Hypertension	13(21.3)	35(16.4)	
Diabetes	4(3.3)	5(2.3)	
Hypertension and diabetes	2(6.6)	14(6.5)	
Initial positive urine culture, n(%)	20(32.8)	44(20.6)	0.046*
Pre-stenting, n(%)	4(6.6)	16(7.5)	0.807
Grade of hydronephrosis, n(%)			0.037*
None	10(16.4)	46(21.5)	
Mild	40(65.6)	151(70.6)	
Moderate	4(6.6)	11(5.1)	
Severe	7(11.5)	6(2.8)	
S-UAS size(Fr), n(%)			0.745
12/14	59(96.7)	205(95.8)	
11/13	2(3.3)	9(4.2)	
Flexible ureteroscope size(Fr), n(%)			0.745
8.5	59(96.7)	205(95.8)	
7.5	2(3.3)	9(4.2)

**Table 3:** Types and proportion of bacteria in initial urine culture before operation

Variable	Infection group(*n* = 61)	Non-infection group(*n* = 213)
Initial positive urine culture, n, (%)	20(32.8%)	44(20.6%)
Urease producing pathogens, n	3	4
Staphylococcus epidermidis, n	1	0
klebsiella pneumoniae, n	1	0
Proteus mirabilis, n	1	0
Pseudomonas aeruginosa, n	0	1
Enterobacter cloacae, n	0	1
Citrobacter freundii, n	0	1
Corynebacterium striatum, n	0	1
Non urease producing pathogens, n	17	40
Escherichia coli, n	7	12
Streptococcus angina, n	1	4
Gardnerella vaginalis, n	4	6
Lactobacillus coil, n	1	1
Citrobacter Coriolis, n	0	1
Candida glabrata, n	0	2
Corynebacterium nonfermentatum, n	0	1
Enterococcus faecalis, n	1	4
Streptococcus agalactiae, n	0	1
Corynebacterium amabilis, n	0	1
Corynebacterium aureus, n	0	1
Streptococcus mitis, n	1	1
Candida tropicalis, n	0	1
enterococcus faecium, n	0	1
Streptococcus gallinolyticus, n	0	1
Corynebacterium glucoliquefaciens, n	1	1
Lactobacillus jensenii, n	1	1

### Postoperative outcomes

The immediate SFR demonstrated comparable outcomes between groups, with 90.2% (62/69) in the infection group and 92.1% (195/212) in the non-infection group (*p* = 0.638). Follow-up evaluations at 3 months revealed no changes in SFR for either cohort. Residual stones were documented in 6 cases from the infection group: 2 localized in the lower pole calyces with concurrent severe hydronephrosis, and 2 intra-diverticular stones measuring < 6 mm. In the non-infection group, 17 patients had residual stones, including 6 lower pole calyceal stones, 4 cases with moderate-to-severe hydronephrosis, and 2 intra-diverticular calculi. No statistically significant differences were observed in postoperative complications, including SIRS, septic shock, subcapsular hematoma, or urinary extravasation. The length of hospital stay also showed no intergroup variation (Table 4). No patients underwent repeat RIRS or additional adjuvant interventions.

**Table 4:** Primary and secondary outcomes

Variables	Infection group(*n* = 61)	Non-infection group(*n* = 213)	*P*-value
Immediately SFR, n(%)	55(90.2)	197(92.1)	0.638
3 months SFR, n(%)	55(90.2)	197(92.1)	0.638
Operative time(min)	72.16 ± 35.75	70.06 ± 29.43	0.639
Degree of ureteral wall injury, n(%)			0.866
None	59(96.7)	206(96.3)	
Grade I	2(3.3)	8(3.7)	
Post-op hospital stays(days)	2[1,2]	2[1,2]	0.866
Second-stage RIRS, n(%)	0(0)	0(0)	-
SIRS, n(%)	2(3.3)	4(1.9)	0.506
Subcapsular haematoma, n(%)	1(1.6)	2(0.9)	0.640
Urinary extravasation, n(%)	1(1.6)	2(0.9)	0.640
Septic shock, n(%)	0(0)	1(0.5)	0.593
Stone compostion			-
Struvite	3	-	
Carbonate apatite	58	-	
Calcium oxalate	-	178	
Calcium phosphate	-	1	
Uric acid	-	32	
Cystine	-	2	
Xanthine	-	1	

### Stone composition analysis

In the infection group, carbonate apatite constituted the majority of stones (95.1%, 58/61), followed by 3 cases of struvite (magnesium ammonium phosphate hexahydrate, 4.9%). The non-infection group was composed predominantly of calcium oxalate stones (83.6%, 178/213), with smaller proportions of uric acid (15.0%, 32/213), cystine (0.9%, 2/213), calcium phosphate (0.5%, 1/213), and adenine stones (0.5%, 1/213).

## Discussion

The formation and growth of infectious stones were closely associated with bacterial presence, and persistent bacterial colonization further complicated treatment [4]. Pathogenic bacteria induced highly alkaline urine and elevated urinary ammonia concentrations, disrupting glycosaminoglycans that protected urothelial cells from bacterial invasion [10]. Bacterial biofilms formed on the mucosal surfaces of the renal pelvis and calyces reduced antibiotic efficacy and promoted antimicrobial resistance. Studies identified common bacteria in infectious stones, such as *Proteus mirabilis*, *Escherichia coli*, and *Klebsiella pneumoniae*, based on stone culture results [4]. In our study, the initial bacterial culture positivity rate was higher in the infectious stone group than in the non-infectious group, though without statistical significance. *E.coli* predominated in the infectious group (35%, 7/20), while *P. mirabilis* accounted for only 5% (1/20). This discrepancy might have stemmed from our limited sample size and reliance on urine culture results with lower sensitivity. The internal biofilm of infectious stones harbored extremely high bacterial loads. These stones were not mere mineral deposits but complex structures composed of bacterial biofilms and crystalline matrices [11]. Notably, bacterial distribution was highly heterogeneous, with dormant populations residing in hypoxic microenvironments within deeper stone layers [12]. Consequently, even with standardized antimicrobial therapy, complete eradication of intrastone bacteria remained unattainable.

The “blast effect” induced by intraoperative laser lithotripsy, as previously described in literature, represents a clinically relevant consideration in stone surgery [13]. This phenomenon, whereby lithotripsy may release bacteria, bacterial debris, and endotoxins from stone matrices, has been associated with potential infection risks—given that stone fragments post-lithotripsy have been shown to harbor bacterial loads that may exceed preoperative urine culture levels [13]. These microorganisms and their byproducts might enter the bloodstream directly through damaged renal pelvic mucosa or via pyelovenous backflow, or disseminate through perirenal lymphatic pathways [14]. Additionally, excessive irrigation pressure (> 30 mmHg) during surgery could force bacteria and toxins into the bloodstream, while negative-pressure suction systems significantly mitigated this risk [15]. Clinical data indicated a 3–5% intraoperative sepsis rate for RIRS without negative-pressure management, whereas negative-pressure technology reduced this risk to < 1% [7]. In our study, no cases of sepsis occurred in the infectious group, and the non-infectious group had only a 0.5% incidence. The negative-pressure flexible ureteroscope maintained continuous low-pressure irrigation but required constant monitoring of outflow patency. As non-intelligent pressure control platforms lacked automatic irrigation cessation, surgeons had to observe fluid circulation in the visual field and immediately retract the scope while stopping irrigation if obstruction occurred. Stone culture was not performed due to laboratory constraints, limiting direct evidence of intrastone bacterial load. Future studies should include stone culture to clarify the link between intrastone bacteria and post-lithotripsy infection risk.

Traditional flexible ureteroscope sheaths exhibited poor reflux efficiency, relying on slow irrigation flow and basket retrieval for limited stone fragment removal, with most fragments dependent on postoperative spontaneous passage [7]. Residual stones served as nidi for recurrence and infection, enabling rapid regrowth, which emphasized the critical importance of immediate postoperative SFR [3]. The negative-pressure ureteroscope system employed a flexible distal suction sheath to rapidly evacuate fragments via hydrodynamic forces. Our study achieved 90.2% immediate SFR in the infectious group, with no change at 3-month follow-up. Analysis of residual stone cases revealed two located in lower calyces with moderate-to-severe hydronephrosis. The expanded renal pelvis during irrigation prevented UAS tip advancement to lower calyces, while mucosal apposition in contracted pelvicalyceal systems increased retention risk. Thus, preoperative feasibility assessment remained crucial for patients with moderate-to-severe hydronephrosis, even with negative-pressure RIRS. Two other residual cases involved diverticular stones with narrow infundibula, no hydronephrosis, infection, or malignancy, warranting conservative management. Due to the limited follow-up duration, conclusions regarding long-term recurrence reduction remained elusive.

Stone physicochemical properties influenced surgical outcomes, including operative time and renal pelvic pressure. Magnesium ammonium phosphate hexahydrate stones, commonly observed in infections, contained an abundant organic matrix and exhibited poor friability [16]. During lithotripsy, debris mixed with pus in obstructed calyces necessitated initial pus clearance to minimize endotoxin absorption. Carbonate apatite stones (95.1% in our cohort), although hard, generated substantial dust during fragmentation, impairing visualization and increasing the risk of ureteroscope-ureteral access sheath (UAS) entrapment, thereby elevating pelvic pressure. Regular scope withdrawal every 1–2 min, fragment clearance, optimized irrigation flow, and enhanced suction were essential for maintaining visualization.

Postoperative surveillance and adjuvant therapy were critical for reducing recurrence. A daily fluid intake of 2–2.5 L, culture-guided antibiotics, and urinary acidification (pH < 6.5) enhanced stone solubility [5]. Postoperative acetohydroxamic acid (AHA), a urease inhibitor, demonstrated anti-recurrence efficacy but carried risks of thromboembolism and tremor. While three randomized trials confirmed AHA’s ability to retard stone growth, it could not dissolve existing calculi [3].

Notably, our preoperative baseline analysis revealed a significantly higher proportion of severe hydronephrosis in the infectious stone group than in the non-infectious stone group (*p* = 0.037). This difference was biologically plausible, as infectious stones were more likely to be associated with prolonged obstruction or inflammation, which might have contributed to the development of severe hydronephrosis. Severe hydronephrosis could theoretically have acted as a confounding factor, potentially increasing operative difficulty or promoting intrarenal stasis, which might have influenced infection-related outcomes or SFR. However, our results showed no significant differences in key clinical outcomes—including SFR, infectious complications (SIRS/sepsis), operative time, or hospital stay—between the two groups, suggesting that despite the imbalance in hydronephrosis severity, FANS-RIRS remained effective and safe in both cohorts. Nevertheless, we had to acknowledge the uncertainty introduced by this baseline difference; given the retrospective nature of the study, we had been unable to adjust or balance this variable post-hoc, which might have introduced selection bias, and the potential impact of severe hydronephrosis on long-term outcomes (e.g., stone recurrence) had also remained unaddressed due to our short follow-up period.

This study has inherent limitations, including its retrospective, single-center design and the relatively small sample size of the infectious stone group (*n* = 61), which may restrict statistical power for rare complications such as sepsis. The 3-month follow-up period is insufficient to assess long-term stone recurrence, a critical endpoint for infectious stones given their high recurrence risk. Due to the study’s time frame (November 2023–December 2024), longer follow-up was not feasible; however, future studies should extend follow-up to 12–24 months to evaluate recurrence in infectious stone patients. We strongly recommend prospective, multicenter trials with larger cohorts to validate our findings and further evaluate long-term outcomes.Stone culture was unavailable due to laboratory constraints. Emerging techniques like radiomics and intraoperative image deep learning might enable noninvasive stone composition analysis, facilitating future prospective validation.

## Conclusion

FANS-RIRS for infectious stones demonstrated comparable postoperative infection rates to non-infectious stones, proving safe and effective. However, postoperative antimicrobial stewardship and regular surveillance remained paramount for infectious stone management.

## Data Availability

The data that support the findings of this study are available from the corresponding author upon reasonable request.
